# KMDATA: a curated database of reconstructed individual patient-level data from 153 oncology clinical trials

**DOI:** 10.1093/database/baab037

**Published:** 2021-06-26

**Authors:** Geoffrey Fell, Robert A Redd, Alyssa M Vanderbeek, Rifaquat Rahman, Bill Louv, Jon McDunn, Andrea Arfè, Brian M Alexander, Steffen Ventz, Lorenzo Trippa

**Affiliations:** Department of Data Science, Dana-Farber Cancer Institute, 450 Brookline Ave, Boston, MA 02115, USA; Department of Data Science, Dana-Farber Cancer Institute, 450 Brookline Ave, Boston, MA 02115, USA; Clinical Trials and Statistics Unit, Institute of Cancer Research, 123 Old Brompton Road, Sutton, London SW73RP, UK; Department of Radiation Oncology, Harvard Medical School, 25 Shattuck St, Boston, MA 02115, USA; Department of Radiation Oncology, Dana-Farber/Brigham and Women’s Cancer Center, 450 Brookline Ave, Boston, MA 02215, USA; Project Data Sphere, 1204 Village Market Place, Suite 288, Morrisville, NC 27560, USA; Project Data Sphere, 1204 Village Market Place, Suite 288, Morrisville, NC 27560, USA; Department of Data Science, Dana-Farber Cancer Institute, 450 Brookline Ave, Boston, MA 02115, USA; Department of Radiation Oncology, Harvard Medical School, 25 Shattuck St, Boston, MA 02115, USA; Department of Radiation Oncology, Harvard Medical School, 25 Shattuck St, Boston, MA 02115, USA; Department of Radiation Oncology, Dana-Farber/Brigham and Women’s Cancer Center, 450 Brookline Ave, Boston, MA 02215, USA; Department of Data Science, Dana-Farber Cancer Institute, 450 Brookline Ave, Boston, MA 02115, USA; Department of Biostatistics, Harvard T.H. Chan School of Public Health, 677 Huntington Ave, Boston, MA 02115, USA; Department of Data Science, Dana-Farber Cancer Institute, 450 Brookline Ave, Boston, MA 02115, USA; Department of Biostatistics, Harvard T.H. Chan School of Public Health, 677 Huntington Ave, Boston, MA 02115, USA

## Abstract

We created a database of reconstructed patient-level data from published clinical trials that includes multiple time-to-event outcomes such as overall survival and progression-free survival. Outcomes were extracted from Kaplan–Meier (KM) curves reported in 153 oncology Phase III clinical trial publications identified through a PubMed search of clinical trials in breast, lung, prostate and colorectal cancer, published between 2014 and 2016. For each trial that met our search criteria, we curated study-level information and digitized all reported KM curves with the software *Digitizelt*. We then used the digitized KM survival curves to estimate (possibly censored) patient-level time-to-event outcomes. Collections of time-to-event datasets from completed trials can be used to support the choice of appropriate trial designs for future clinical studies. Patient-level data allow investigators to tailor clinical trial designs to diseases and classes of treatments. Patient-level data also allow investigators to estimate the operating characteristics (e.g. power and type I error rate) of candidate statistical designs and methods.

**Database URL**: https://10.6084/m9.figshare.14642247.v1

## Introduction

The magnitude and form of treatment effects in cancer clinical trials (e.g. early, delayed or persistent improvements of survival probabilities (1) relative to the standard of care) can vary substantially across malignancies, patient populations and classes of anti-cancer therapeutics (1, 2). This makes the design and analysis of cancer clinical trials with time-to-event primary outcomes challenging. Collections of survival data from completed clinical trials and real-world datasets can support the choice of study designs and statistical procedures for future clinical studies (3, 4). In particular, datasets from completed trials can enable the estimation and comparison of operating characteristics (e.g. power, type I error rate and the risk of exposing patients to inferior treatments) of various study designs and statistical methods (e.g. proportional hazards and accelerated failure-time models). Importantly, study designs and statistical methods can be evaluated with retrospective analyses that focus on a specific disease (5, 6) or on a class of treatment (7).

Individual patient-level data (IPLD) from completed clinical trials can support key decisions in future studies. These data can inform the selection of suitable metrics to quantify treatment effects (e.g. difference in median survival, or the restricted mean survival). For example, the hazard ratio (HR) can be difficult to interpret in settings where, based on previous experience, one expects delayed treatment effects on overall survival (OS) (8). IPLD can also help predict the operating characteristics of candidate data-analysis procedures. Rahman *et al.* (1) and Uno *et al.* (9) showed the utility of leveraging context-specific data from completed clinical trials to select suitable data-analysis techniques. Retrospective analyses of survival data from completed trials can complement or replace the use of arbitrarily selected simulation scenarios to compare designs and methodologies (10). Finally, IPLD can be used for planning interim analyses and decisions during the trial. For example, Ventz *et al.* (11). discussed non-inferiority designs based on a collection of head and neck cancer datasets and provided recommendations on interim monitoring procedures for future head and neck cancer trials.

Although several important initiatives have facilitated access to IPLD from completed clinical trials (12–14), access to survival data from clinical studies remains limited, and typically it does not include recent trials. Additionally, in our experience, data repositories require research proposals and time-consuming procedures to obtain access to clinical trial datasets.

We created a database of reconstructed IPLD that includes individual time-to-event outcomes, such as OS and progression-free survival (PFS). These outcomes were extracted from Kaplan–Meier (KM) survival curves (15) reported in 153 Phase III oncology studies in breast, lung, prostate and colorectal cancer, published between 2014 and 2016 in eight major oncology journals: *Annals of Oncology*, *Lancet, Lancet Oncology*, *Journal of the American Medical Association (JAMA)*, *JAMA Oncology*, *Journal of Clinical Oncology*, *Journal of the National Cancer Institute* and *New England Journal of Medicine.* We discuss small discrepancies between our estimates of the IPLD outcomes and those in the actual datasets. Importantly, our reconstructed KM and IPDL are directly available and downloadable by interested users. Moreover, our database can be used for comparisons of statistical designs and methodologies.

## Database

### Inclusion criteria

**Figure 1.**
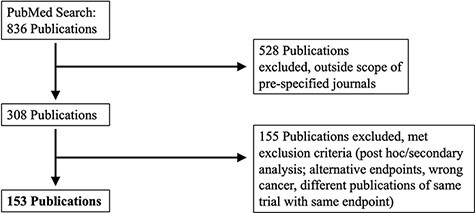
PRISMA selection of clinical trials included in KMDATA. The original PubMed search resulted in 836 total publications. Results were excluded based on prespecified criteria including cancer type and study endpoints. A total of 153 publications from clinical trials were included in the database.

A PubMed search was performed on 4 December 2017. MeSH search terms included ‘*breast cancer*’, ‘*lung cancer*’, ‘*prostate cancer*’ or ‘*colorectal cancer*’, and results were limited to Phase III clinical studies, published between 1 January 2014 and 31 December 2016 in eight major clinical journals: *Annals of Oncology*, *Lancet, Lancet Oncology*, *Journal of the American Medical Association (JAMA)*, *JAMA Oncology, Journal of Clinical Oncology*, *Journal of the National Cancer Institute* and *New England Journal of Medicine.* We further restricted results to clinical studies with tumor-directed interventions that reported at least one time-to-event outcome with KM curves. Time-to-event outcomes included OS, PFS, disease-free survival and relapse-free survival. Figure 1 summarizes our search and inclusion/exclusion criteria.

The PubMed search identified 153 published clinical trials. The identified manuscripts included 304 KM graphs that compared the distributions of time-to-event outcomes in the experimental and control arms. We call these graphs ‘KM pairs’. For clinical trials with multiple experimental arms and a control group, we defined ‘KM pairs’ for each experimental treatment. The average sample size per treatment arm was 432 (SD = 448, range = 31–2661). For each KM pair, we extracted and curated study-level information on the cancer type, publication date, journal, trial registration ID, PubMed ID, type of intervention, type of experimental therapy, trial population, trial design, randomization ratio, sample size, primary endpoint(s), reported HR and *P*-values for the primary endpoints.

#### Reconstructed IPLD

For each of the 153 studies that met our search criteria, all KM survival curves were extracted from the publication as a raster image. Next, we obtained the coordinates $\left\{ {\left[ {{t_i}, \hat S\left( {{t_i}} \right)} \right]; i = 1, \ldots , d} \right\}$ of the KM curves with the software *Digitizelt* (16). Here, $\hat S\left( {{t_i}} \right) $indicates the KM estimate of the survival function at event times ${t_i}$, $i = 1, \ldots , d$. Patients-at-risk tables are often included at the bottom of KM graphs. We extracted these patients-at-risk tables $\left\{ {\left( {{{\tilde t}_i}, {r_i}} \right); i = 1, \ldots , D} \right\}$ from the publications. In OS analyses, ${r_i}$ indicates the number of patients with survival larger than ${\tilde t_i}$; more generally, in time-to-event analyses, ${r_i}$ is the number of patients at risk at time ${\tilde t_i}$.

Next, we used the KM survival curve $\{ ({t_i}, \hat S\left( {{t_i}} \right); i = 1, \ldots , d\} $, together with the patients-at-risk table $\left\{ { \left( {{{\tilde t}_i}, {r_i}} \right); i = 1, \ldots , D} \right\}$ if it was available, to estimate patient-level time-to-event outcomes with censoring $\left\{ { \left( {{T_j}, {C_j}} \right); j = 1, \ldots , n} \right\}$. Following the standard notation of time-to-event analyses, $j = 1, \ldots , n $indexes patients and ${C_j} \in \left\{ {0,1} \right\}$ indicates if the time variable ${T_j}$ corresponds to an observed event (${C_j} = 1, $ e.g. the patient’s death in OS analyses) or to a censored event (${C_j} = 0, $ e.g. patient $j$ was alive at the end of a follow-up period of ${T_j}$ months in OS analyses). We estimated the individual variables $\left( {{T_j}, {C_j}} \right)$ with the algorithm proposed by Guyot *et al.* (17).

### Data structure

**Figure 2.**
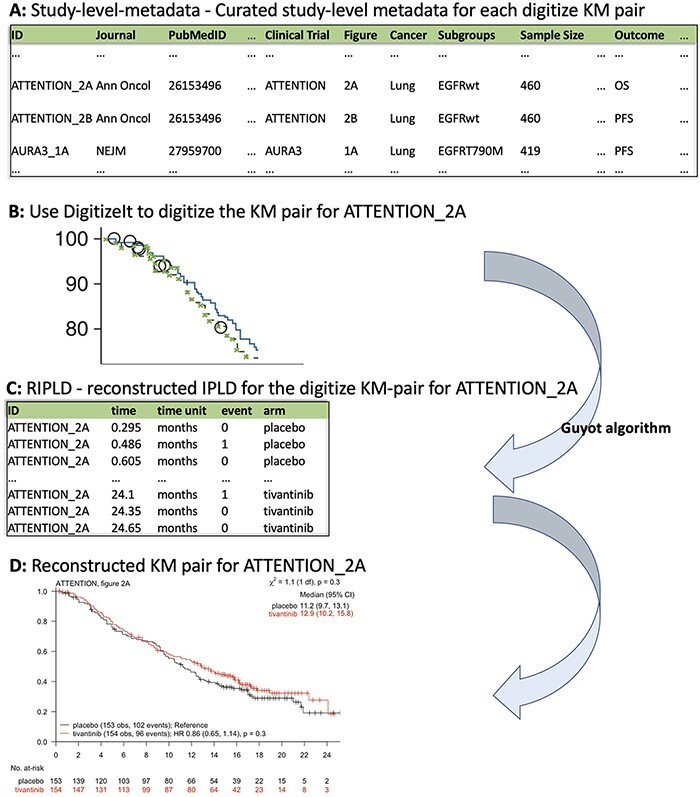
KMDATA structure. Each publication (A) was digitized (B) and processed using the Guyot algorithm to obtain time-to-event reconstructed data (C). The digitalized figures are included in the ***kmdata*** package. (D) Example of the reconstructed figures.

The KMDATA database is available in two forms: an excel file (MASTER.DATA.xlsx), available from 10.6084/m9.figshare.14642247.v1), or an R package (*kmdata)*, available on GitHub at https://github.com/raredd/kmdata (18). Both formats include study-level metadata, demographic information and the reconstructed IPLD (Figure 2). The R package also implements the Guyot algorithm (17) for users interested in reconstructing IPLD from KM images using the *ipd* function.

#### Study-level metadata

This table contains 23 variables for each KM pair (Figure 2A). These variables include the *PubMed-ID* of the publication from which we extracted the KM pair, the reported sample size of the study, the cancer type, the type of intervention and the primary outcome. The variable *ID* links the KM pairs in the study-level metadata table and the reconstructed IPLD table (RIPLD).

Multiple entries (rows) of the study-level metadata table may be dedicated to each clinical study. Indeed, clinical studies typically report KM curves for multiple endpoints (OS, PFS, etc.) and in various patient subpopulations. These entries have the same *PubMed-ID* and clinical-trial identifiers, while the variables *Subgroups* and *Outcome* indicate the subpopulation and the endpoint described by the KM pair. The variable *Figure* identifies the figure and panel of the article from which we extracted the KM pair, *Units* indicates the time scale (weeks, months or years from enrollment), *Treatment-Arms* indicates the control and experimental treatments and *Intervention-Class* classifies the experimental treatments (categories include chemotherapy, immunotherapy, targeted therapy and surgical intervention).

#### Example

The first entry in Figure 2A corresponds to a KM pair from Figure 2A (figure) of the lung cancer (cancer) study ATTENTION (clinical trial) published in Annals of Oncology (Journal). The reported sample size of ATTENTION was 460 (sample size) patients, and the KM pair describes the OS (*Outcome*) outcome in the Epidermal Growth Factor Receptor (EGFR)-wildtype (subgroups) subpopulation.

#### Demographics

This table contains 12 variables. It reports, for each clinical study (publication) and treatment arm (therapy), demographic information of the enrolled patients, such as the number of male and female patients (sex:males, sex:females) and the median age at enrollment (median age).

#### Reconstructed IPLD

The RIPLD table contains, for each of the 304 KM pairs, the reconstructed (possible censored) patient-level time-to-event outcomes. This table has five columns: ID, time, time unit, event and arm. The variable *ID* (first column in both study-level metadata and RIPLD tables) provides a unique identifier to link the KM pairs listed in the study-level metadata table to the reconstructed time-to-event outcomes in the RIPLD file. For example, all RIPLD rows with *ID* ATTENTION_2A correspond to the OS KM curves (the *Outcome* variable for the ATTENTION_2A column in the study-level metadata file) in Figure 2A of Yoshioka *et al.*, 2015 (19) for the ATTENTION trial.

The variables *time* and *event* in the RIPLD file provide the reconstructed event-times ${T_j}$ and censoring indicators$ {C_j}.$ Also, the variable *time unit* (weeks, months or years from enrollment) indicates the unit of measure of ${T_j}$ and the variable *arm* assigns the reconstructed outcome (${T_j}$, ${C_j}$) to the experimental or the control arm.

#### Example

The first entry in the *RIPLD* table in Figure 2C, (i.e. *ID:* ATTENTION_2A, *time*: 0.295, *time unit*: months, *event*: 0, *arm:* placebo) is a reconstructed OS outcome from the publication of the *lung cancer* study *ATTENTION* (19). It refers to an EGFR-wildtype patient randomized to the *placebo* arm with censored OS-time $({C_j} = 0)$ after ${T_j} = 0.295$ months from randomization.

### Validation analysis

We computed several summary statistics from our *RIPLD* and compared them to those reported in the publications. We focused on four measures:

- The estimated HR between the experimental and control treatments, using a univariate Cox proportional hazards model,
- The median event-time in each treatment arm,
- The number of events and
- The number of patients at risk, as reported in the patients-at-risk tables.

For each of these summaries, the scatterplots in Supplementary Figure S1 show data summaries reported in the publications (*x*-axis) against the summaries computed with the RIPLD (*y*-axis). Points close to the diagonal line indicate matched and recovered data summaries, whereas points far from the diagonal line indicate datasets with RIPLD summaries that deviate from the published results.

The table *ts2* included in KMDATA (in the Excel file MASTER.DATA.xlsx) indicates, for each of the four summary measures, the absolute (*Abs.Diff*) and relative (*Rel.Diff*) difference between published and reconstructed data summaries. These discrepancy measures can be used to filter KM pairs with limited agreement between reconstructed and published data summaries, according to user-specified criteria.

### Validation analysis based on datasets from Project Data Sphere (PDS)

We identified six trials (NCT00703326, NCT00785291, NCT00981058, NCT00988208, NCT00988208 and NCT01193244) in the KMDATA database that were available for download from PDS (20), a data repository of completed clinical trials. For all six datasets, IPLD were only available for the control arms of the study. We randomly selected three of them (NCT00703326, NCT00785291, NCT00981058) and compared the actual IPLD from PDS and our reconstructed IPLD. The comparison is based on KM graphs computed using (i) the actual IPLD from PDS or (ii) our reconstructed IPLD from the digitalized publications. For the ROSE study (NCT00703326), the version of the dataset from PDS and the version reported in Mackey *et al.* (21), as well as the corresponding follow-up periods, are different.

Supplementary Figure S2 illustrates that the KM curves in black (actual IPLD data) and blue (reconstructed IPLD data) are almost identical for the CALGB40502 (21) (NCT00785291) and SQUIRE (22) (NCT00981058) studies. As expected, for the ROSE study (NCT00703326) we observe some discrepancies between the black and blue KM curves. These are due to different follow-up periods and versions of the dataset presented in Mackey *et al.* (21) (the digitalized manuscript) and in the PDS platform.

### Usage notes

The KMDATA database can be accessed for data analyses with R (23) (package ***kmdata)***. We illustrate an example here. We consider OS in the lung cancer study ACT1 (PMID25794890). We illustrate how to compare the experimental and control arms with a log-rank test, estimate the HR between these two arms and graph the reconstructed data.

*\(# install the kmdata package\)* *\(# install.packages(\)\!\!\!\!‘\(devtools\)’\!\!\!\!)*devtools::install_github(\!\!\!\!‘raredd/kmdata’\!\!\!\!, \, build_vignettes=TRUE) library(\!\!\!\!‘kmdata’\!\!\!\!)

KM.pairs= ls(\!\!\!\!‘package:kmdata’\!\!\!\!)length(KM.pairs) *\(# number of KM pairs\)* ## [1] 308

KM.pairs[1:5]    *\(# first 5 objects of the\)\, database* ## [1] "ACT1_2A" "ACT1_3A" "ACT2_2A"\, "ACT2_2B" "ACT2_2C" 000

Next, we print the first entries of the OS RIPLD for *ACT1(*Figure 2A*in PMID25794890).* Use help(ACT1_2A) and attributes(ACT1_2A) to obtain information about the reconstructed IPLD for Figure 2A of ACT1, including trial-level information and quality scores for the reconstruction.

head(ACT1_2A)    *\(# look at first entries\)* ##   time event      arm## 1 0.210   0 amrubicin## 2 0.336   1 amrubicin## 3 0.336   1 amrubicin 000

We then use the RIPLD and a log-rank test to compare the OS survival distributions of the experimental (amrubicin) and control (topotecan) arms.

S = Surv(ACT1_2A$time, ACT1_2A$event) *\(# create\)\, \(\, survival object\)*survdiff(S ~ ACT1_2A$arm)             *\(# log\)-\(rank\)\, \(\, test\)* ## Call:## survdiff(formula = S \(\sim\) ACT1_2A$arm)####      N Observed Expected (O-E)^2/E (O-E)\(\widehat{\phantom{x}}\)2/V## ACT1_2A$arm=topotecan 213 175 161 1.306 1.94## ACT1_2A$arm=amrubicin 424 333 347 0.603 1.94#### Chisq= 1.9 on 1 degrees of freedom, p= 0.2 000

Last, we fit a Cox proportional hazards model to estimate the HR and use the function **kmplot()** to plot the reconstructed KM curves.

summary(coxph(S \(\sim\) ACT1_2A$arm))$coefficients ##         coef exp(coef) se(coef) z Pr(>|z|)## ACT1_2A$armamrubicin -0.1307436 0.87744280.09359198 -1.396953 0.1624278 kmplot(ACT1_2A) 000

#### Note

After installing the ***kmdata*** package from github (https://github.com/raredd/kmdata), a detailed explanation of the ***ipd()*** function, which reconstructs the IPLD, is available via **help(topic = ‘ipd’)**. The data-frame ***kmdata_key*** in ***kmdata*** contains the *Study-level metadata* table, use **head(kmdata_key)** to print the first six entries of the table. The R code to reproduce Figure S1 is available as a vignette in the ***kmdata*** package.

## Supplementary Material

baab037_Supp
