# Supplementary material for: KMDATA: a curated database of reconstructed individual patient-level data from 153 oncology clinical trials
Source: Database (Oxford). 2021 Jun 26;2021:baab037. doi: 10.1093/database/baab037 (PMC8234134; doi:10.1093/database/baab037)

Supplementary Material for: "KMDATA: A Curated Database of Reconstructed Individual Patient-Level Data from 153 Oncology Clinical trials"

Authors: Geoffrey Fell*, Robert A Redd*, Alyssa M. Vanderbeek, Rifaquat Rahman, Andrea Arfè, Brian M. Alexander, Steffen Ventz**, Lorenzo Trippa**

* Co-first author

** Co-senior author

Department of Data Science, Dana-Farber Cancer Institute, Boston, MA (GG, RAR, AA, SV, LT)
Harvard T.H. Chan School of Public Health, Boston, MA (SV, LT)
Harvard Medical School, Boston, MA (RR, AA, BMA)
Department of Radiation Oncology, Dana-Farber/Brigham and Women's Cancer Center, Boston, MA (RR, BMA)
Clinical Trials and Statistics Unit, Institute of Cancer Research, Sutton, UK (AMV)

Figure S1: Data summaries estimated from reconstructed IPLD were compared to the results reported in each publication. Metrics used include (A) number of patients at-risk at fixed time points; (B) total number of events (e.g., deaths); (C) hazard ratios; and (D) arm-specific median time-to-event. The published and reconstructed summaries are shown on the x-axis and y-axis, respectively.

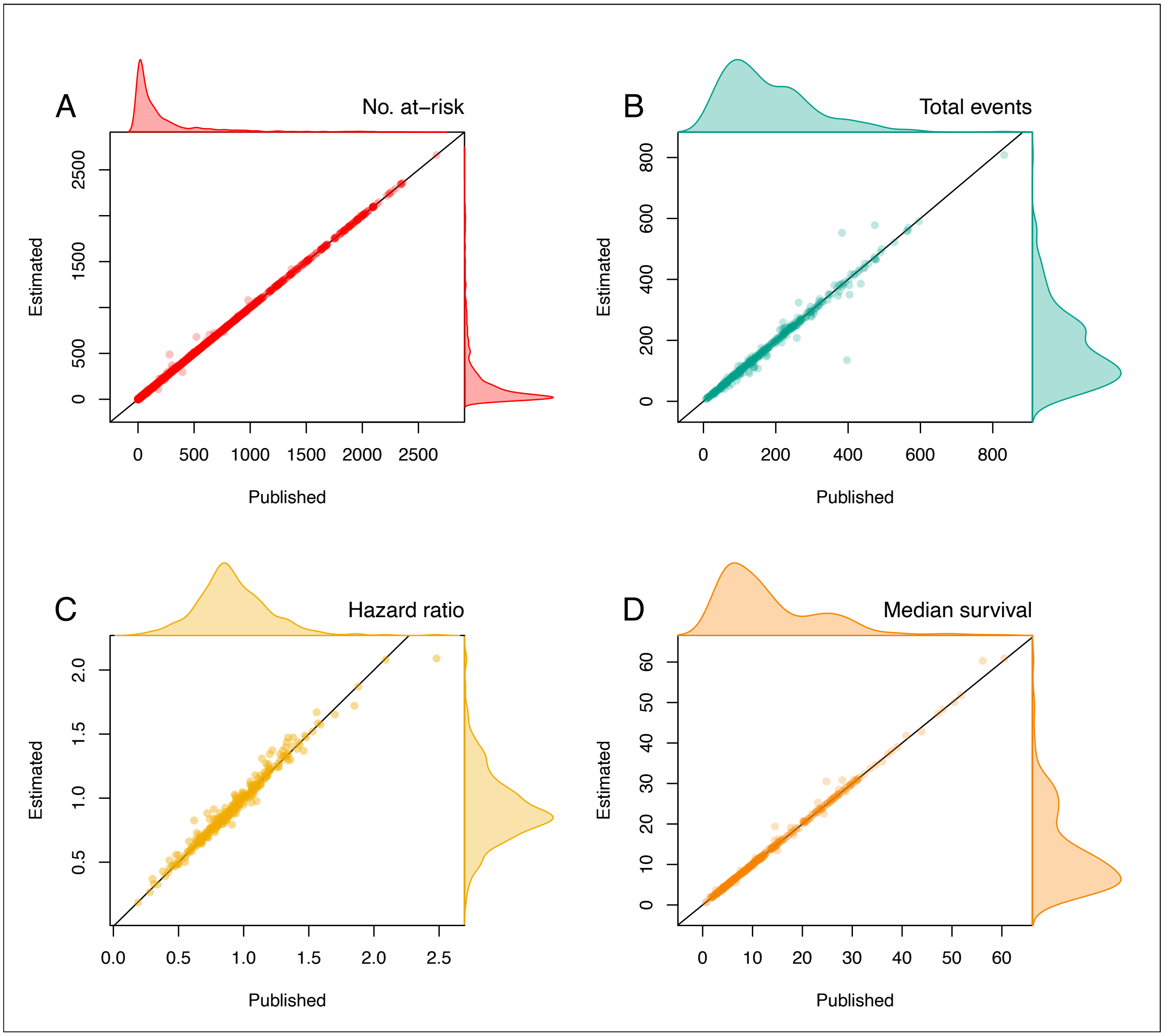

Figure S2: Comparison of the actual IPLD obtained from PDS (control arms only) and our reconstructed IPLD. Each panel illustrates KM estimates computed using (i) the actual IPLD from PDS (black) or (ii) our reconstructed IPLD from digitalized publications (blue). The title of each panel indicates the clinical trial (CALGB40502, ROSE and SQUIRE) and the Figure/Panel in the articles (Mackey et al[2], Mehrotra et al.[4] and Thatcher et al.[5]) that we digitalized to reconstruct IPLD.

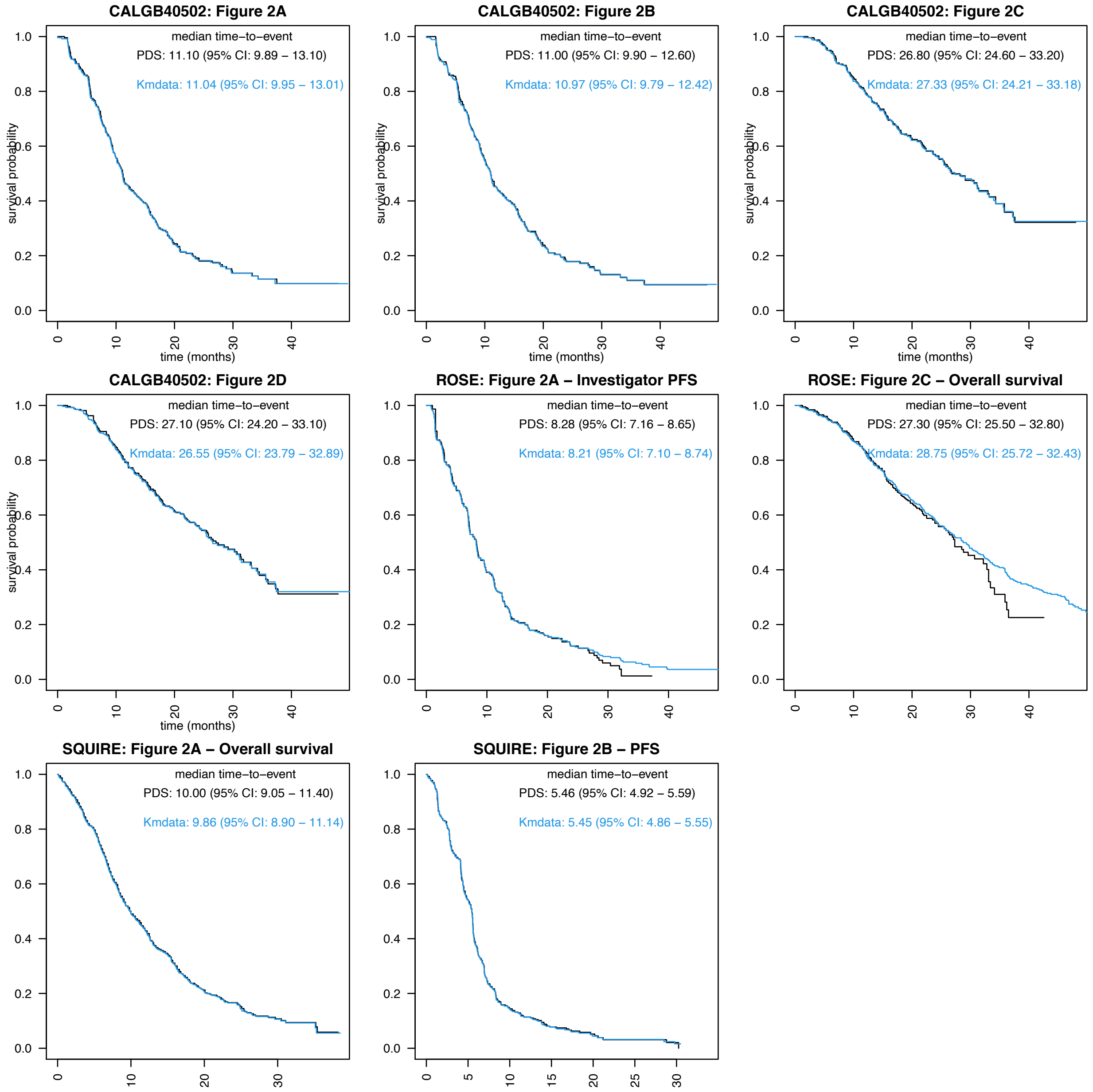

Supplement: baab037_Supp [file baab037_supp.zip › Supplementary Material.pdf]
